# Swimming prevents cell death of chondrocytes via PI3K/AKT pathway in an experimental model

**DOI:** 10.1186/s13018-023-03815-4

**Published:** 2023-06-17

**Authors:** Jiajia Qian, Peiru Zhao, Qi Xu, Weiwei Yang, Ren Cai

**Affiliations:** 1 Department of Rehabilitation Therapy, Nanjing University of Chinese Medicine, Nanjing, Jiangsu China; 2 Laboratory for New Techniques of Restoration & Reconstruction of Orthopedics and Traumatology, Nanjing University of Chinese Medicine, Nanjing, Jiangsu China; 3 Department of Basic Physical Education, Nanjing University of Chinese Medicine, Nanjing, Jiangsu China

**Keywords:** Osteoarthritis, Swimming, Autophagy, Apoptosis, PI3K/AKT

## Abstract

**Background:**

Knee Osteoarthritis (KOA) is one of the main causes of disability in the elderly and with limited treatment options. Swimming was considered as an ideal form of non-surgical management of KOA. Nevertheless, the mechanism of swimming intervene OA remains unclear. ACLT induced OA model was often used to study the pathogenesis and treatment of OA. Thus, we evaluated the protective effect of swimming on KOA mouse and tried to explore the underlying mechanism.

**Methods:**

Forty C57BL/6 mice were randomly divided into five groups: Blank group, ACLT group, ACLT + Swim group, Sham group and Sham + Swim group (*n* = 8). OA model was established by Anterior Cruciate Ligament Transection surgery (ACLT). After modeling, mice in ACLT + Swim and Sham + Swim groups were trained with a moderate swimming program, 5 d/week, for 6 weeks. HE and Safranin-O/fast staining, Immunohistochemistry, TUNEL assay and Western blot were used to detect the effect of swimming on pathological changes, cell death and the mechanism in KOA mouse.

**Results:**

Swimming significantly enhanced CoII expression and suppressed ADAMTS5 expression in cartilage of KOA mouse, thus ameliorated KOA development. Apoptotic and autophagic processes were enhanced in OA cartilage, which might be caused by down-regulation of PI3K/AKT pathway; swimming could activate PI3K/AKT pathway and thus regulate apoptosis and autophagy processes of chondrocytes.

**Conclusion:**

Swimming could prevent cell death of chondrocytes via PI3K/AKT pathways, thus delayed the progression of KOA in an experimental model.

**Supplementary Information:**

The online version contains supplementary material available at 10.1186/s13018-023-03815-4.

## Introduction

Osteoarthritis (OA) is one of the most costly and disabling forms of chronic arthritis, representing a major public health burden [1]. OA causes pain, deformity, reduction of motion, and then affect daily activities, particularly among the elderly [2–4]. However, to date, there were no effective therapies can completely prevent the progression of OA attributed to the limited understanding of exact molecular mechanisms of OA [5]. Chondrocytes were the only cell type existing in healthy cartilage; therefore, any perturbation of the chondrocytes can afflict OA progression [6]. Researchers suggested the term ‘‘Chondroptosis’’ to describe death of chondrocytes, which include the presence of some apoptotic and autophagic processes [7]. Therefore, strategies aiming at the inhibition of cell death hold promise in slowing down the progression of OA.

The American College of Rheumatology suggested that OA therapeutic strategies should include aerobic exercise [8]. Swimming was an ideal aerobic exercise for middle-aged and elderly patients with OA. Many studies have confirmed that swimming can improve the mobility, muscle strength and functionality of patients with KOA, and delay the progress of OA [9–12], but the mechanism of swimming intervene OA remains unclear. In the present study, whether swimming can inhibit cell death by regulating autophagy and apoptosis of chondrocytes in KOA mouse was explored. In addition, the molecular mechanism of swimming on osteoarthritis was investigated by researching changes of the phosphoinositide 3‑kinase (PI3K)/AKT signaling pathway.

## Materials and methods

### Animal model establishment

Forty C57BL/6 mice (12 week-old) of both sexes were purchased from Shandong skobas Biotechnology Co, Ltd (Shandong, China). The mice were adaptively fed with food and water with free access for 1 week; subsequently, mice were randomly divided into three groups: Blank group (*n* = 8), ACLT group (*n* = 16), Sham group (*n* = 16). In the ACLT group, we established a surgically induced moderate OA model by Anterior Cruciate Ligament Transection surgery (ACLT) in the right leg of mouse, the surgical protocol was described in previously researches [13, 14]. In the sham group, only a 1.5 cm incision in the same position was made, but the ligament was not cut.

### Experimental design

Two weeks after the surgery, mice in ACLT group and Sham group were further divided into the following groups: ACLT, ACLT + Swim, Sham, Sham + Swim. As an innate ability of rodents, swimming exercise presents advantages over treadmill running [15]. Mice were trained with a moderate swimming program according to the report of Liu et al. [15]: Swimming program included two phases: adaptation and training phase. In the first week for adaptation, swimming beginning with 15 min on the first day to 60 min on the last day. The adaptation exercise was aimed at reducing the water-induced stress without promoting physiological alterations in relation to the physical training [16]. Then, mice swam for 60 min/day, 5 days/week, for a total of 5 weeks. The animals were swum as a group of four to six mice, because it has been demonstrated that the intensity of swimming exercise was significantly raised by interaction among the animals [17]. Swimming was continuously supervised. Experiments performed in this study were all approved by the Animal Experiment Committee of Nanjing University of Chinese Medicine (Ethics No: 201904A009).

### Hematoxylin–eosin (HE) and Safranin-O (S-O)/fast staining

Knee tissues of each group (*n* = 3) were fixed in 4% paraformaldehyde for 24 h, then de-calcified in 10% EDTA for 8 weeks. After dehydrated in graded ethanol, tissues were embedded in paraffin. Then, 4 μm sections were stained with Hematoxylin–eosin and Safranin-O/fast Green. Three to five fields were randomly selected from each section and observed under a 200 × light microscope. The pathological changes were evaluated thrice and graded by 3 independent researchers. Then, we used the Osteoarthritis Research Society International (OARSI) (0–12) score to estimate the degree of articular cartilage destruction [18].

### Immunohistochemistry

Knee joint sections of each group (*n* = 3)(4 μm) were mounted on glass slides and subjected to immunohistochemistry staining. After antigen repaire, 1% bull serum albumin was used to seal tissue for 20 min. The primary antibody was anti-ADAMTS5 (1:100, Bioss) and anti-COL-II (1:100, Affinity). Tissue sections were incubated with first antibody overnight. Then goat anti-rabbit secondary antibody was added and incubated for 20 min. DAB chromogen was used to visualize antibody labelling. The protein expression was observed under a light microscope, and three areas with high expression were taken and photographed for storage (all pictures were taken at 400X).

### Terminal deoxynucleotidyl transferase dUTP nick end labeling (TUNEL) staining

Cartilage tissues of each group (*n* = 3) were fixed in paraformaldehyde and prepared in paraffin sections. 3% H2O2-methanol solution was used for 10 min rinsing. The slices were then incubated with 0.2% Triton for 5 min, followed by two times of PBS rinsing. Following the manual instructions, 50μL TUNEL reaction mixture (KGA702China) was added into each tissue slice, followed by 37 °C dark incubation. After three times of PBS rinsing, images were taken under a fluorescent microscope.

### Western blot

Proteins of knee cartilage from each group (*n* = 3) were extracted and collected using RIPA Lysis Buffer (KGP250, China), mixed with 10 μl phosphatase inhibitor, 1 μl protease inhibitor and 5 μl 100 mM PMSF. Protein concentrations of each group were determined by BCA kit (KGA902, China). Then, electrophoresis was performed, blocking with 10% milk powder for 2 h, incubated with primary antibodies BAX (diluted1:1000, abcam ab32503), BCL-2 (diluted1:2000, abcam ab182858), Cleaved-caspase3 (diluted1:200, abcam ab214430), Cleaved-caspase9 (diluted1:1000, CST 9509 T), Beclin-1 (diluted1:1000, abcam ab210498), LC3II/LC 3I (diluted1:2000, abcam ab192890), p-PI3K (diluted 1:500, UK abcam ab182651), PI3K (diluted 1:1000, UK Abcam ab191606), p-AKT (diluted 1:5000, abcam ab81283), AKT (diluted 1:10,000, abcam ab179463). After washing again, secondary antibodies were added and incubated for 2 h at room temperature. Image-J software was used to analysis the Gray scale.

### Statistical analysis

The results were displayed as mean ± SD. All data were analyzed with GraphPad Prism 8 statistical software. Differences among three groups were analyzed by one-way analysis and *p* < 0.05 indicated statistical significance.

## Results

### Swimming ameliorated OA development in the ACLT mouse model

**Fig. 1:**
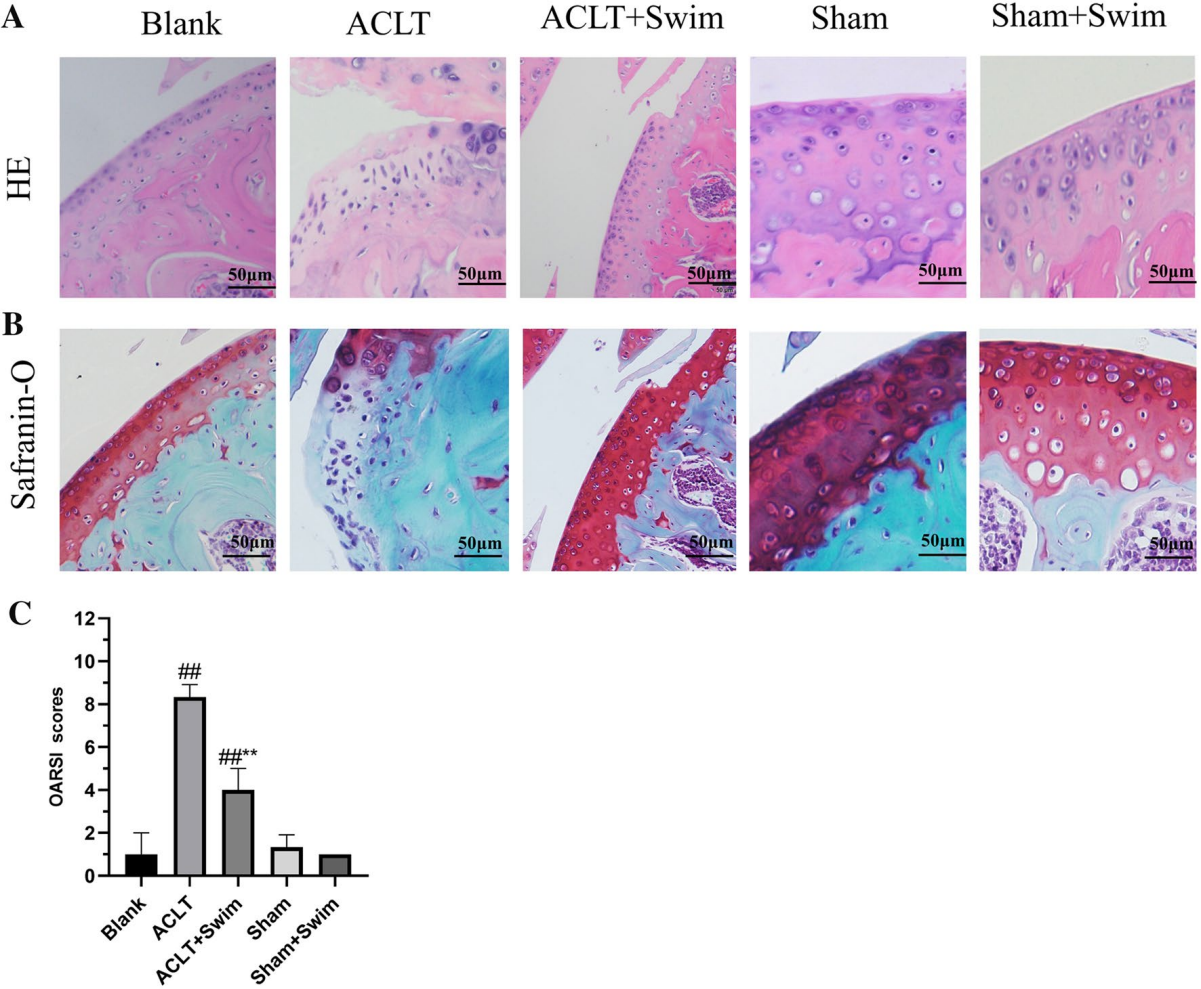
Histopathological analysis of the cartilage tissues obtained from each group (scale bar: 50 μm) to show the development of OA. **A**: After fixation, decalcification and embedding, 4 mm frontal sections were cut from the knee joints and were stained with HE staining. **B**: Safranin-O/fast green staining images of cartilage in each group. **C**: OARSI score. The data in the figures represent mean values ± SD, #*p* < 0.05, ##*p* < 0.01 compared with the Blank group, *p < 0.05, **p < 0.01 compared with the ACLT group

Histological analysis of cartilage tissue was performed using HE and Safranin-O/fast green staining. As shown in (Fig. 1A and B), the cartilage surface exhibited a positive red stain; the surface was smooth and intact in the Blank group and Sham group. However, the model groups exhibited cartilage superficial destruction, a high proteoglycan loss and apparent hypocellularity. Nevertheless, moderate swimming could mitigate these detrimental effects; it remarkably protected the structure of articular cartilage and maintained the proteoglycan in cartilage. Next, we performed OARSI score on cartilage in different groups (Fig. 1C). The scores of the OA group were clearly higher than those of the sham control group (*p* < 0.01). In contrast, swimming group showed transparently lower OARSI scores than the ACLT group (*p* < 0.01). Altogether, these results indicate that swimming attenuates the development of OA in the ACLT mouse model.

### Swimming enhanced ECM synthesis and reduced ECM degradation in cartilage

**Fig. 2:**
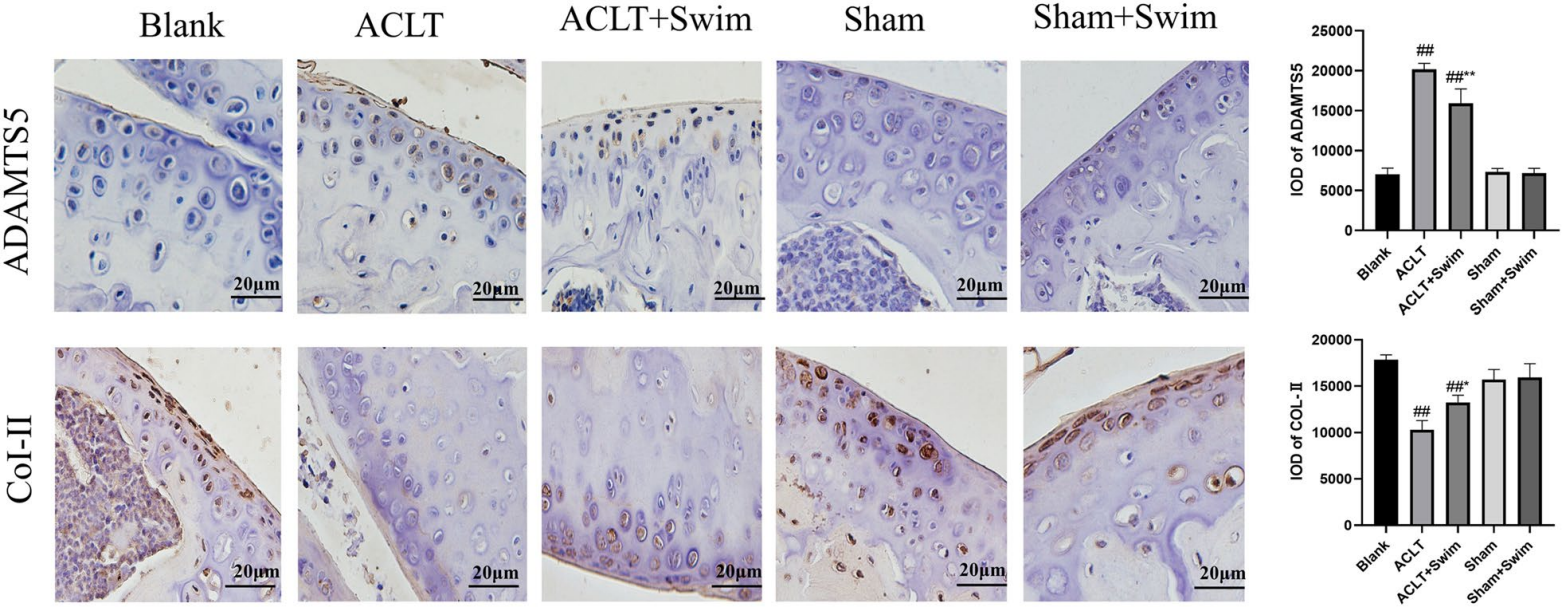
Swimming shows a protective effect on the ECM metabolism. The expression of cartilage matrix biomarkers ADAMTS5, COL-II of knee joint was detected by Immunohistochemistry staining (scale bar: 20 μm). The data in the figures represent mean values ± SD, #*p* < 0.05, ##*p* < 0.01 compared with the Blank group,*p < 0.05, **p < 0.01 compared with the ACLT group

Next, we determined the effect of swimming on ECM synthesis and degradation of cartilage by investigating the expression of CoI-II and ADAMTS5 by Immunohistochemistry. As Fig. 2 show, the expression of ADAMTS5 increased and resulting in decrease of CoI-II in ACLT group. But the up-regulation of ADAMTS5 in model mice were inverted by swimming. On the contrary, expression of CoI-II was improved after 6 weeks swimming evidently. Taken together, these results indicate that swimming alleviates ECM degradation and promotes ECM synthesis in OA.

### Swimming inhibited apoptosis of chondrocyte

**Fig. 3:**
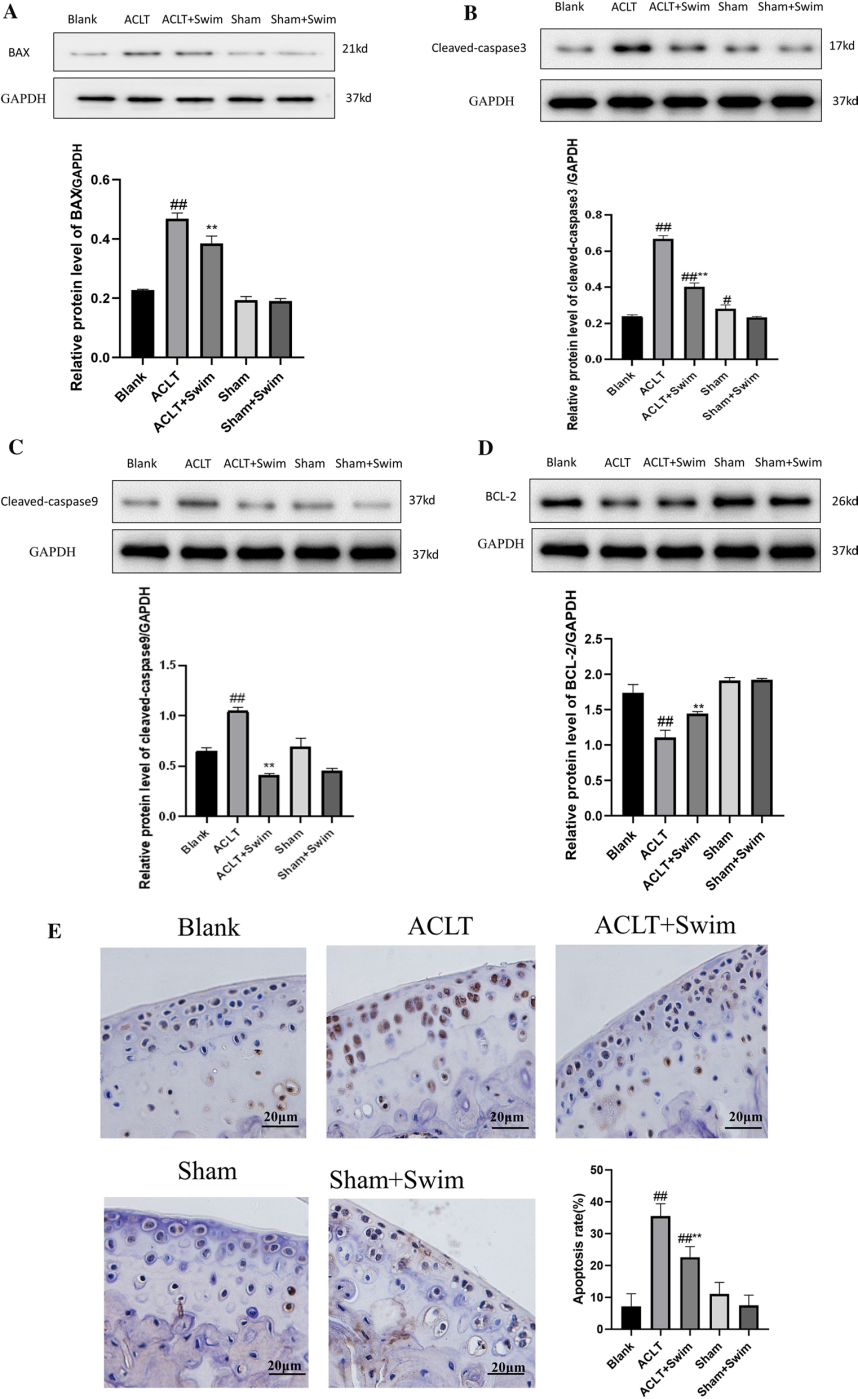
Effect of swimming on inhibiting apoptosis of chondrocyte. **A**–**D**: protein expression of Bax, cleaved-caspase3, cleaved-caspase9 and Bcl-2in the cartilage of OA model; **E**: apoptosis rate of each group observed by TUNEL staining. The data in the figures represent mean values ± SD, #*p* < 0.05, ##*p* < 0.01 compared with the Blank group, *p < 0.05, **p < 0.01 compared with the ACLT group

The effect of swimming on cell apoptosis of chondrocytes was analyzed using Western blot and TUNEL staining. The results showed that compared with the Blank group, protein expression of Bax, cleaved-caspase3 and cleaved-caspase9 in the cartilage of OA model increased markedly, and the expression of Bcl-2 decreased significantly, while 6 weeks swimming significantly decreased Bax, cleaved-caspase3 and cleaved-caspase9, and increased Bcl-2 protein expression when compared to the ACLT group (Fig. 3A–D) (*p* < 0.01).

According to the TUNEL staining (Fig. 3E), the apoptotic rate of model group was much higher (35.53% ± 3.87%) compared with Sham and Blank groups (*p* < 0.01), with thinner regional cartilages and less cells. The apoptotic cartilages were mainly located in the middle layer and on the outer surface of the cartilage in OA. While in ACLT + Swim group, apoptotic rate declined significantly (22.62% ± 3.30%, *p* < 0.01). All together, these indicating that swimming could inhibit the apoptosis of chondrocytes in OA.

### Swimming inhibited autophagy of chondrocytes

**Fig. 4:**
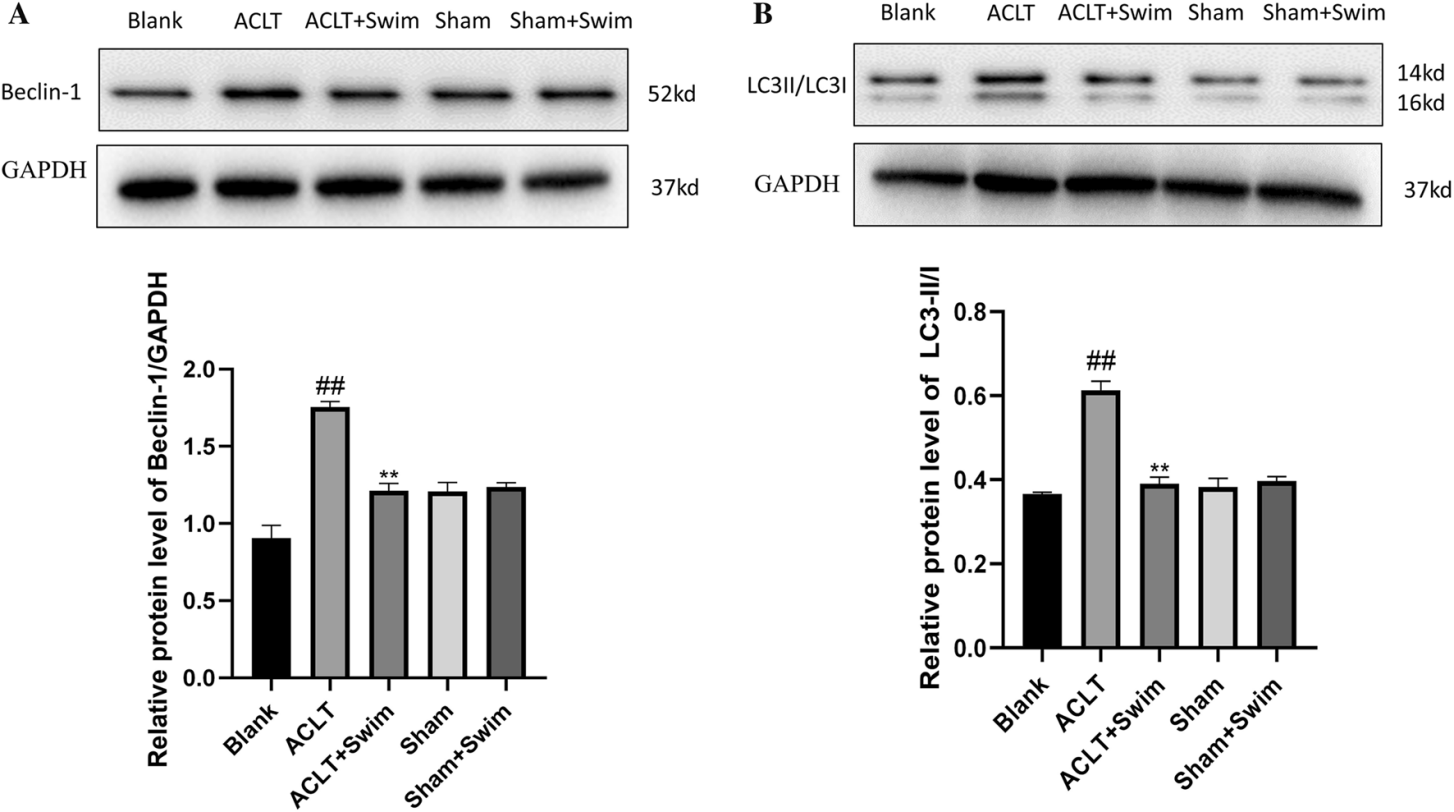
Effect of swimming on inhibiting autophagy of chondrocyte. Relative expression of autophagy-related proteins LC3II/LC3I and Beclin-1 from mouse cartilage evaluated by Western Blotting. The data in the figures represent mean values ± SD, #*p* < 0.05, ##*p* < 0.01 compared with the Blank group, *p < 0.05, **p < 0.01 compared with the ACLT group

Samples from cartilage of each group were used to identify Beclin-1 and LC3II/LC3I (autophagy markers) by Western blot (Fig. 4A and B). A basal expression of Beclin-1 and LC3II/LC3I was observed in normal cartilage of Blank group and Sham groups. In OA pathogenesis Beclin-1 and LC3II/LC3I expression increased, the results were consistent with the previous studies; autophagy could be conjunctly activated with apoptosis as an alternative pathway to cellular death [19]. While in the swimming group, a significant decrease in autophagy markers was observed. This suggested that swimming could also inhibit the autophagy of chondrocytes in OA.

### Swimming promoted activation of the PI3K/AKT pathway

**Fig. 5:**
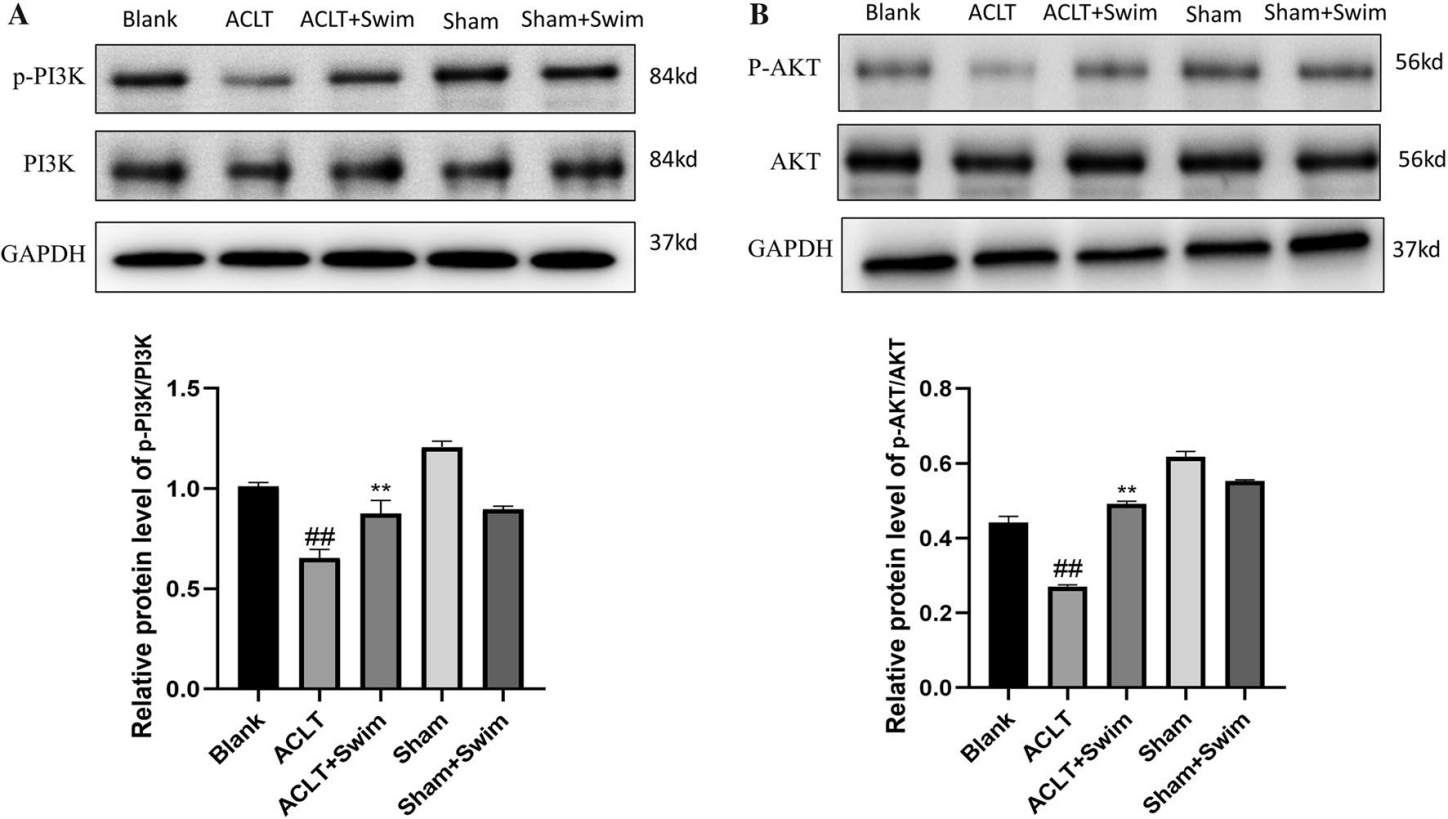
Effect of swimming on PI3K/AKT activation in OA cartilage. The protein expression levels of P-PI3K, PI3K, P-AKT, AKT, and GADPH were determined by western blot and quantification analyses. The data in the figures represent mean values ± SD, #*p* < 0.05, ##*p* < 0.01 compared with the Blank group, *p < 0.05, **p < 0.01 compared with the ACLT group

To further investigate the protection mechanism of swimming on OA, the effects of swimming on PI3K/AKT phosphorylation were detected by western blot analysis. As shown in Fig. 5, the phosphorylation of PI3K and AKT in ACLT group significantly down-regulated compared to the Blank group. However, moderate swimming activatedPI3K/AKT phosphorylation (Fig. 5). The results demonstrated that swimming could activate P13K/AKT signaling pathway thus suppressed apoptosis and autophagy of chondrocytes.

## Discussion

With the aging of the population and rising obesity, OA has become a major problem for health systems globally [4]. Knee OA is the most common arthritis and one of the main causes of disability in the elderly [20]. Current treatments of OA can be divided into surgical and non-surgical [4]. Osteoarthritis is a progressive disease; in the late stage, no treatments are effective enough to delay the development except surgical procedure: joint replacement [1, 21]. According to the OARSI guidelines (2019) of non-surgical management of OA, most therapies were mainly emphasizing on the prevention and symptom relief, such as habit adjustments (e.g., weight loss, regular exercises) and symptomatic treatment by drugs (e.g., Nonsteroidal anti-inflammatory drugs) [22]. The American College of Rheumatology suggests that aerobic exercise should be included in OA treatment [8]. Swimming is a common aerobic exercise, since the buoyancy of water enables patients to bear minimal weight-bearing stress, and the duration and load of exercise are in control, made swimming an ideal form of aerobic exercise for middle-aged and older patients with OA. Previous researches already demonstrated that swimming can improve the symptoms of patients with KOA [9–12], and can alleviate cartilage degradation in rats [23]. But the underlying molecular mechanism was still unknown.

Mouse model is now the most ideal animal model for the study of molecular backgrounds of physiological and pathological conditions [13]. The anterior cruciate ligament transection (ACLT) surgery induced OA model was often used to study the pathogenesis and treatment of OA [24]. Previous studies have shown that ACLT model was very similar to human OA, including subchondral changes, articular cartilage damage and synovitis [25]. Consequently, in the current study, we aimed to explore the protective mechanism of swimming on osteoarthritis based on ACLT induced KOA mouse model.

Chondrocytes are the only cells present in cartilage, which strongly involved in maintaining the dynamic equilibrium between synthesis and degradation of the extracellular matrix (ECM). And the death of chondrocytes and the loss of ECM are the key features in cartilage degeneration during OA pathogenesis [26]. Research has been documented that matrix metalloproteinases (MMPs, especially MMP-13) and a disintegrin and metalloproteinase with thrombospondin motifs (ADAMTSs, especially ADAMTS4 and ADAMTS5) facilitate CoI-II and aggrecan degradation, respectively [27]. Our previous research has confirmed that significant expression of ADAMTS5 was observed in the OA mouse [28]. Roach et al. proposed the term “Chondroptosis” to suggest the type of cell death in articular cartilage, which included some elements of classical apoptosis and autophagy [7, 29]. Chondrocyte apoptosis was an important feature in OA cartilage, release of various inflammatory factors contributes to apoptosis of chondrocytes [30], thus lead to progressive degeneration of OA [31, 32]. Therefore, inhibition of cell apoptosis after OA may be the key to promote chondrocyte repair [33]. In this study, the results showed that swimming had the effect of improving the progress of osteoarthritis and inhibiting the chondrocytes apoptosis after OA.

Autophagy is regarded as a survival mechanism; generally, it plays an important role in maintaining cellular homeostasis, and its dysregulation has been linked to non-apoptotic cell death [34–36]. In the pathogenesis of OA, apoptosis and autophagy are considered as two important links. Now, it is believed that autophagy as an adaptive response can reduce cell death in early stage of OA, but with the development of OA, excessive autophagy may also cause cell death [37]. Autophagy is regulated by a series of autophagy-related genes (Atg), such as Beclin1and LC3. Beclin1 can form a complex with type III phosphatidylinositol 3-kinase and Vps34 thus allows nucleation of the autophagic vesicle [38]. LC3 has 2 forms; during autophagy, LC3-I is converted to LC3-II through lipidation by a ubiquitin-like system, resulting in the association of LC3-II with autophagy vesicles. So, the amount of LC3-II is correlated with the extent of autophagosome formation [39]. Our researches showed that in OA mouse, autophagy was also activated, which caused cell death together with apoptosis, and swimming can actually inhibited autophagy in ACLT mouse.

Many pathways involved in the cell death; PI3K/AKT signaling pathway is a vital pathway to regulate cell cycle, apoptosis and proliferation [40]. Studies have shown that PI3K is required for the differentiation and survival of chondrocytes in normal growth plate in vitro and therefore is also necessary for the growth in cartilage [41]. It is demonstrated that the inhibition of the P13K/AKT signaling pathway contributes to the mechanisms termed apoptosis and autophagy of chondrocytes [42, 43]. After the activation of PI3K, inositol can be phosphorylated, which promotes the activation of AKT directly or indirectly Phosphorylated-AKT is the main form of the activation of AKT [44]. P-AKT can inhibit apoptosis by suppressing the activity of FKHR, NF-kappa B and GSK-3, inhibiting phosphorylation of Bad, caspase-9 and inhibiting the release of apoptosis factors by mitochondrial [45]; it can also stimulate the TOR/P70S6‐kinase pathway to suppress the autophagy in mammalian cells [46]. Taken together, this research showed that swimming can activate P13K/AKT pathway; this may be the underlying mechanism of inhibition of chondrocyte death (Additional file 1).

## Conclusion

In conclusion, this study showed that moderate swimming exercise slowed the development of KOA in ACLT mouse; moreover, this research also demonstrated that the Chondroptosis (apoptosis and autophagy) in cartilage of KOA mouse can be inhibited by swimming exercise, which might be associated with activating the PI3K/AKT signaling pathway. Hoping these findings can provide experimental support for the research of KOA therapeutic strategies.

## Supplementary Information

**Additional file 1:** Original western blots.

## Data Availability

The datasets used and/or analyzed during the current study are available from the corresponding author on reasonable request.

## Abbreviations

PI3K/AKT: Phosphoinositide 3‑kinase/Akt
OA: Osteoarthritis
ECM: Extracellular matrix
ACLT: Anterior cruciate ligament transection surgery
HE: Hematoxylin–eosin
S-O: Safranin-O
ADAMTS5: A disintegrin and metalloproteinase with thrombospondin motifs 5
COL-II: Type II collagen
TUNEL: Terminal deoxynucleotidyl transferase dUTP nick end labeling
OARSI: Osteoarthritis Research Society International
