# Supplementary material for: Swimming prevents cell death of chondrocytes via PI3K/AKT pathway in an experimental model
Source: J Orthop Surg Res. 2023 Jun 17;18:439. doi: 10.1186/s13018-023-03815-4 (PMC10276397; doi:10.1186/s13018-023-03815-4)
Supplement: Supplementary file 1 — Additional file 1: Original western blots. [file 13018_2023_3815_MOESM1_ESM.pptx]

## Slide 1
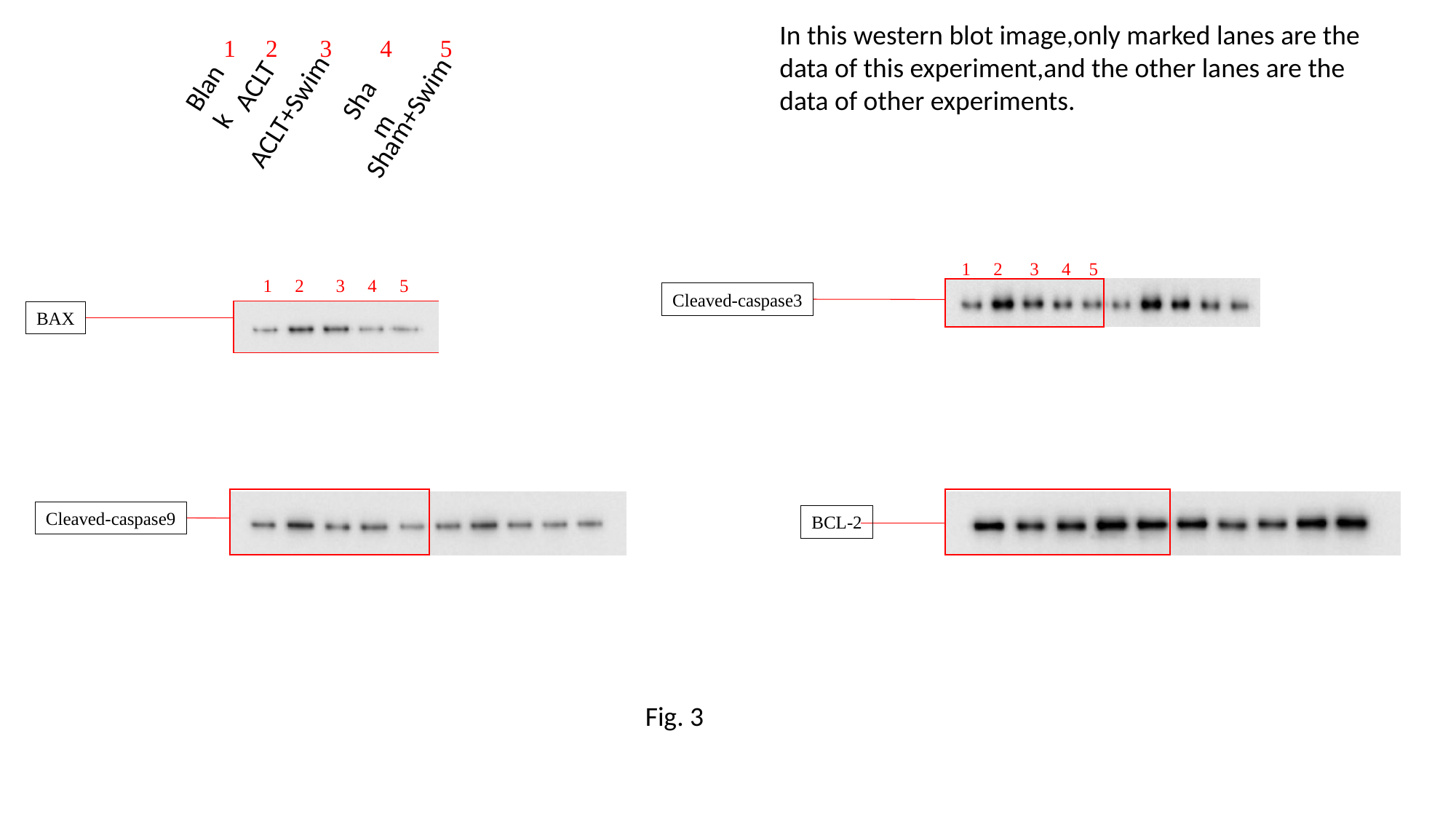

In this western blot image,only marked lanes are the data of this experiment,and the other lanes are the data of other experiments.
ACLT+Swim
1 2 3 4 5
Sham+Swim
Blank
ACLT
Sham
1 2 3 4 5
1 2 3 4 5
Cleaved-caspase3
BAX
Cleaved-caspase9
BCL-2
Fig. 3

## Slide 2
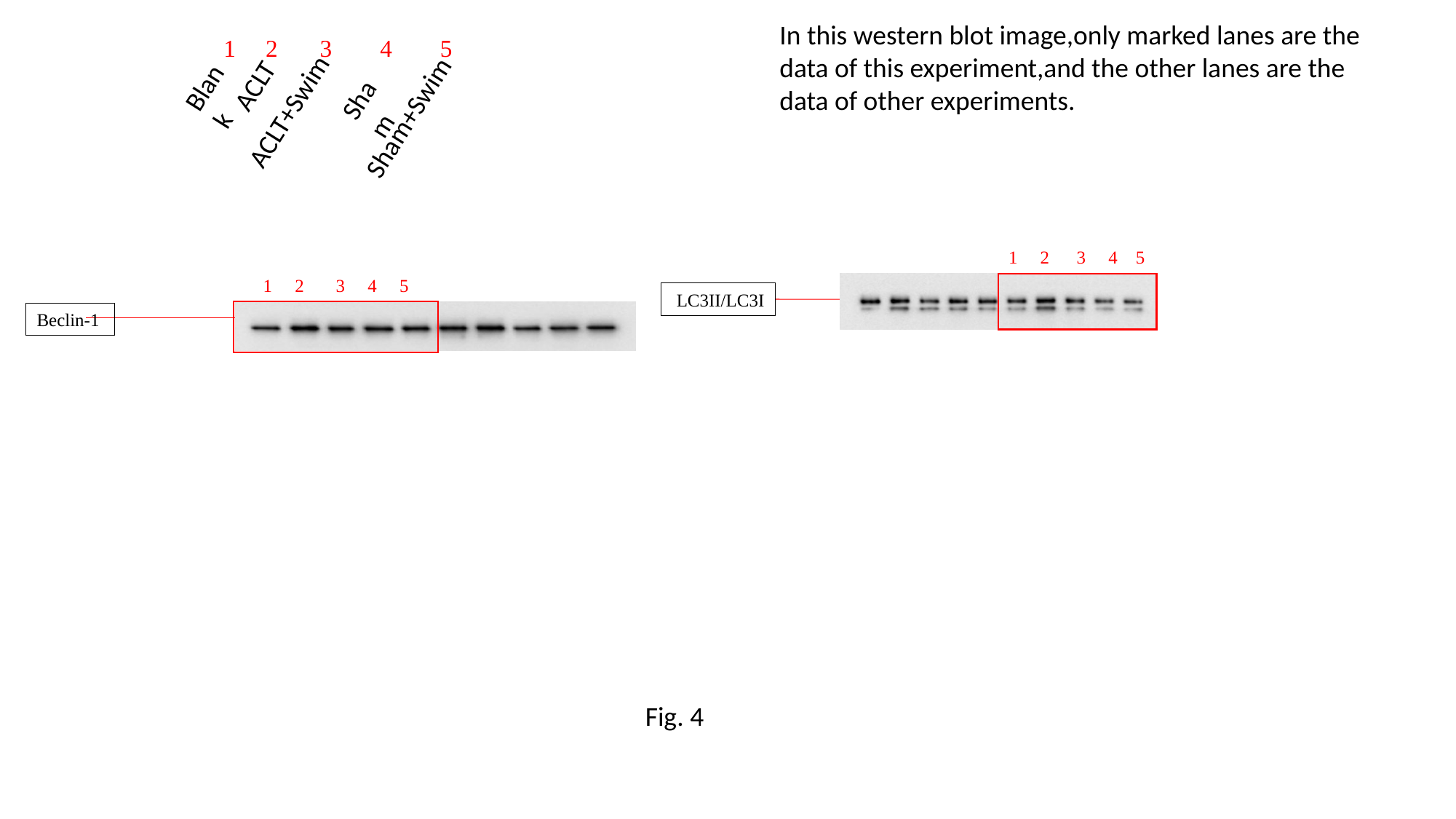

In this western blot image,only marked lanes are the data of this experiment,and the other lanes are the data of other experiments.
ACLT+Swim
1 2 3 4 5
Sham+Swim
Blank
ACLT
Sham
1 2 3 4 5
1 2 3 4 5
 LC3II/LC3I
Beclin-1
Fig. 4

## Slide 3
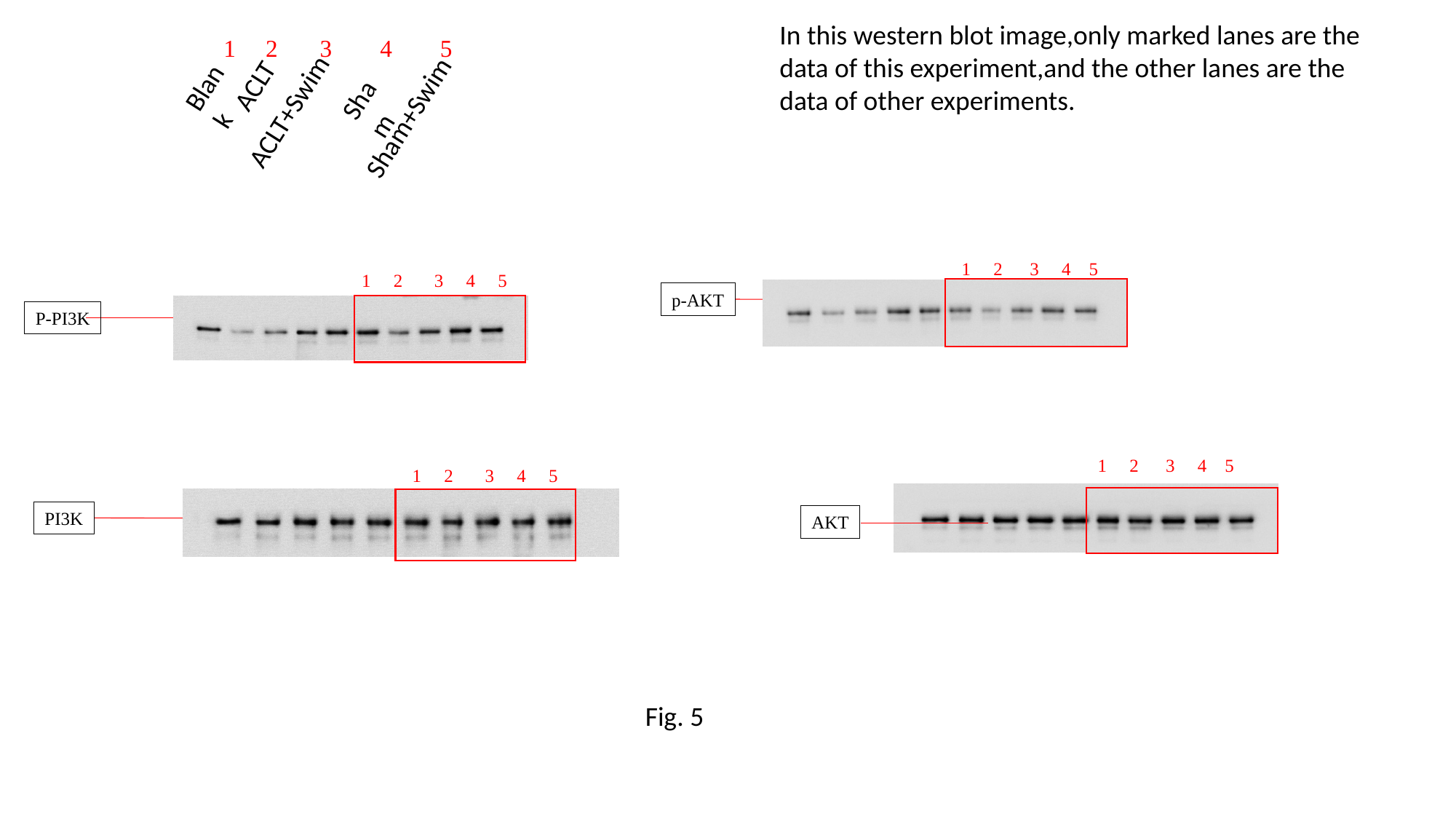

In this western blot image,only marked lanes are the data of this experiment,and the other lanes are the data of other experiments.
ACLT+Swim
1 2 3 4 5
Sham+Swim
Blank
ACLT
Sham
1 2 3 4 5
1 2 3 4 5
p-AKT
P-PI3K
1 2 3 4 5
1 2 3 4 5
PI3K
AKT
Fig. 5
